# Bacterial Infections, DNA Virus Infections, and RNA Virus Infections Manifest Differently in Neutrophil Receptor Expression

**DOI:** 10.1100/2012/527347

**Published:** 2012-03-12

**Authors:** Esa-Matti Lilius, Jari Nuutila

**Affiliations:** Department of Biochemistry, University of Turku, 20014 Turku, Finland

## Abstract

Treating viral illnesses or noninfective causes of inflammation with antibiotics is ineffective and contributes to the development of antibiotic resistance, toxicity, and allergic reactions, leading to increasing medical costs. A major factor behind unnecessary use of antibiotics is, of course, incorrect diagnosis. For this reason, timely and accurate information on whether the infection is bacterial in origin would be highly beneficial. In this paper we will present our recent studies on the expression of opsonin receptors on phagocytes. The analysis of the expression levels of Fc*γ*RI, CR1, and CR3, along with CRP and ESR data, provides a novel application to the diagnosis of infectious and inflammatory diseases. The best clinical benefit will be obtained when the individual variables are combined to generate the CIS point method for a bacterial infection marker, DNAVS point for differentiating between DNA and RNA virus infections, and CRP/CD11b ratio for a marker of Gram-positive sepsis.

## 1. Introduction

There are two basic molecular mechanisms of recognition of microorganisms by phagocytic cells: opsonin-dependent, and opsonin-independent. The former mechanism requires serum components, opsonins, which act by binding to the surface of the microorganisms at one end and to specific receptors on the phagocyte surface at the other. The best-known opsonins are Immunoglobulin G (IgG) which binds via its Fc domain to the Fc receptor (FcR) on the phagocytes, and the C3b and iC3b fragments of the C3 component of complement, which bind to the complement receptors CR1 and CR3, respectively, on the phagocyte surface. Many different types of bacteria interact with phagocytic cells in serum-free media *in vitro* in the absence of opsonins, Certain integrins (CR3 among others) serve as receptors for microbial surface ligands in nonopsonic phagocytosis [1]. 

IgG is the most abundant Ig class in serum, constituting over 75% of circulating immunoglobulin. It mediates key effector functions through interaction with Fc*γ* receptors. Fc*γ* receptors are divided generally into three main classes, Fc*γ*RI(CD64), Fc*γ*RII (CD32), and Fc*γ*RIII (CD16), each with distinct structural and functional properties. Fc*γ*RI is a high-affinity receptor for monomeric IgG (*K*_*a*_: 10^9^–10^10^/M) with three extracellular Ig-like domains expressed constitutively by monocytes and macrophages, as well as by many myeloid progenitor cells. In contrast to Fc*γ*RI, the other two classes of Fc*γ* receptor, Fc*γ*RII and Fc*γ*RIII, display low affinity for monomeric IgG. They are capable of binding to aggregated IgG through multimeric low-affinity, high-avidity interactions, which are particularly important in the recognition and binding of antibody-antigen complexes during an immune response. IgG binding to low-affinity Fc*γ*R can trigger a range of effector and immunoregulatory functions, including degranulation, phagocytosis, and regulation of antibody production. Fc*γ*RII is expressed by diverse cell types: Fc*γ*RIIa isoform by myeloid cells, including polymorphonuclear leucocytes, monocytes, macrophages, platelets, and certain types of endothelial cells and, Fc*γ*RIIb by B cells, monocytes, and macrophages, while Fc*γ*RIIc expression is restricted solely to natural killer (NK) cells. The other low-affinity Fc*γ* receptor, Fc*γ*RIII, is in two isoforms. Although Fc*γ*RIIIa and Fc*γ*RIIIb share high levels of sequence homology, they exhibit distinct structural differences. Fc*γ*RIIIa is a transmembrane protein that associates with the Fc*γ*R chain, whereas Fc*γ*RIIIb is processed posttranslationally as a glycosylphosphatidylinositol- (GPI-) anchored protein, lacking transmembrane and intracellular domains. The Fc*γ*RIIIa isoform is expressed widely by several leucocyte cell types, including macrophages, NK cells, and subsets of T cells and monocytes, while Fc*γ*RIIIb is expressed constitutively only by neutrophils [2].

The receptors for complement molecules, designated complement receptors 1 (CD35) and 3 (CD11b), present on all phagocytic cells, are only weakly expressed on the surface of resting neutrophils and are mostly stored in intracellular granules (CD35 in secretory vesicles and CD11b in both specific and gelatinase granules and secretory vesicles). Secretory vesicles (SV) are the most likely to release contents via degranulation, followed by gelatinase, specific, and azurophil granules. In addition to exposure to proinflammatory cytokines, even relatively simple physical stress, different anticoagulant types, temperature changes, and isolation of leukocytes can trigger rapid degranulation of neutrophil SVs. Fusion of SVs with the plasma membrane leads to increased CD35 and CD11b levels at the cell surface [3]. 

Do the infectious and other inflammatory diseases induce alterations in the expression of the opsonin receptors of phagocytes?

## 2. Receptor Expression Measurements

Erythrocytes were lysed in anticoagulated blood by adding 10 volumes of 0.83%  NH_4_Cl followed by 15 min of incubation at room temperature. Leukocytes were separated by centrifugation. Before the measurements of receptor expression, leukocytes (3 × 10^5^) were incubated in 50 *μ*L of gHBSS with monoclonal antibodies (0.4 *μ*g) in polystyrene flow cytometer vials for 30 min at +4°C. After incubation, the cells were washed once with cold gHBSS and resuspended in cold gHBSS. Leukocytes incubated with mouse unspecific immunoglobulins served as controls for correction of leukocyte autofluorescence. A relative measure of receptor expression was obtained by determining the mean fluorescence intensity of 5000 leukocytes. In the case of neutrophil Fc*γ*RI, the percentage of fluorescence positive cells (%) was also determined. In neutrophils, which express only few Fc*γ*RI (MFI < 4.0) on the cell membrane, the %-value varied between 5 and 70 which described the changes in expression levels better than the MFI. At a high expression levels (MFI value >4.0), the %-value was 95–100. When %-value was 100 regardless of the activation state of leukocyte (i.e., in the case of CR1, CR3, and Fc*γ*RII in neutrophils, monocytes, and eosinophils, Fc*γ*RI in monocytes, and Fc*γ*RIII in neutrophils), only MFI was presented. Measurement of leukocyte receptor expression was performed using fluorescently (FITC or PE) labelled receptor-specific monoclonal antibodies. The receptor panel studied for two-colour immunofluorescence analysis and the mAbs are presented in Table 1.

## 3. Receptor Expression in Health and Diseases 

Earlier, we have performed few studies where we have measured the receptor expression in various rather small patient groups [4–7]. The summary of the results from these studies is presented in Table 2. In monocytes, all the receptors were upregulated in bacterial and viral infections. In neutrophils, CR1, CR3, Fc*γ*RI, and Fc*γ*RII were upregulated, while Fc*γ*RIII was downregulated in bacterial infections. CR1 and Fc*γ*RII were downregulated, while CR3 and Fc*γ*RI were upregulated in viral infections. These results led us to conclude that the receptor expression could be used as a basis for the differential diagnosis of bacterial and viral infections. 

## 4. Prospective Study 

**Figure 1:**
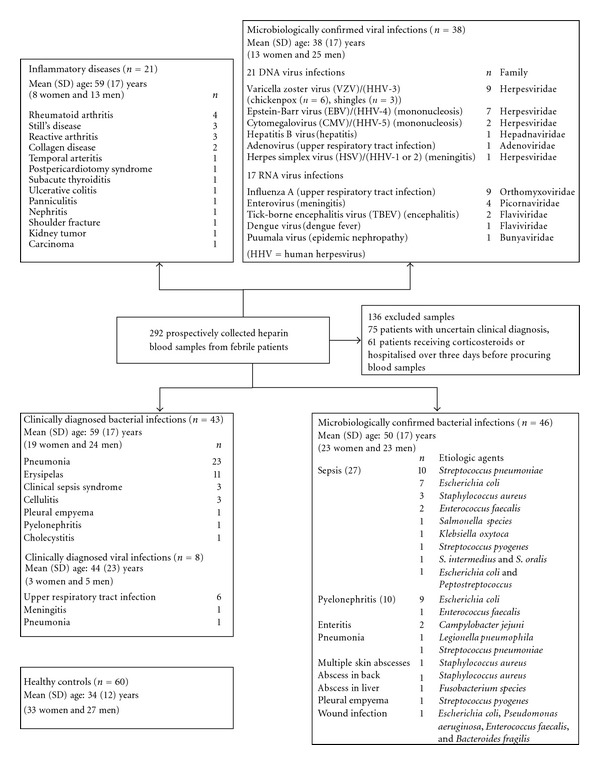
Subgroups of patients. Subgroup classification was based on medical and microbiological examination, including bacterial cultures, serological assays, and identification of microbial antigens or nucleic acids from nasopharyngeal, urine, cerebrospinal fluid, or blister specimens. The healthy volunteer control group is also defined. Parentheses include the number of presented cases.

**Figure 2:**
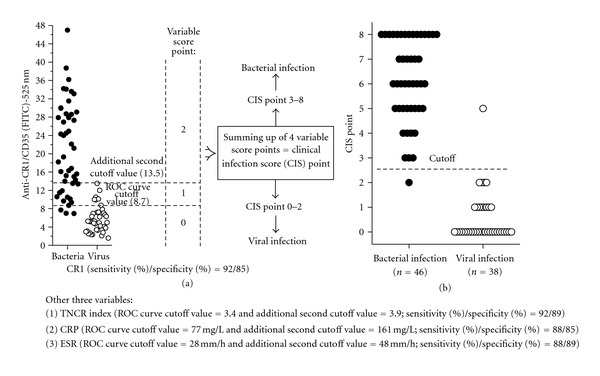
Formation of clinical infection score (CIS) point.

In this study, standard clinical laboratory data (neutrophil count, serum C reactive protein level (CRP), and erythrocyte sedimentation rate (ESR)) and quantitative flow cytometric analysis of neutrophil complement receptors, CR1 and CR3, as well as Fc*γ*RI (CD64) were obtained from 292 hospitalized febrile patients. After microbiological confirmation or clinical diagnosis, 135 patients were found to have either bacterial (*n* = 89) or viral (*n* = 46) infection. The patient data was compared to 60 healthy controls. The grouping of the patients into subgroups is presented in Figure 1. The mean of parameters measured in the patient samples are presented in Table 3. The average expression levels of CR1 and CR3 on neutrophils in bacterial infections were over threefold and twofold higher, respectively, compared with viral infections and controls. According to receiver operating characteristic (ROC) curve analysis, neutrophil CR1 displayed 92% sensitivity and 85% specificity in distinguishing between bacterial and viral infections (Figure 2(a)). Compared with other measured variables, such as neutrophil CR3, neutrophil count, CRP, and ESR, neutrophil CR1 had the most effective differential capacity. The lower diagnostic accuracy of CR3 compared with CR1 may be explained by the phenomenon that CR3 is expressed not only from rapidly releasing secretory vesicles like CR1, but also from specific and gelatinase granules [8]. The differential capacity of CR1 and CR3 was lost when EDTA, instead of heparin, was used as an anticoagulant (Table 3) due to defaults in extracellular calcium in blood samples. The behaviour of CRP and ESR was similar to the expression of neutrophil CR1 in that they were significantly higher in bacterial than in viral infections. In addition to the measured variables, we defined a computational variable by multiplying the neutrophil count, mean fluorescence intensity (MFI) of FITC-conjugated CR1-specific monoclonal antibodies on neutrophils and MFI of PE-conjugated CR3-specific monoclonal antibodies on neutrophils (= neutrophil count × relative number of CR1 on neutrophils × relative number of CR3 on neutrophils). The index obtained by taking the base-10 logarithm of this factorial represents the total number of neutrophil complement receptors per blood sample volume (TNCR index, Table 3.) The TNCR index has somewhat higher specificity (89% versus 85%) than neutrophil CR1 in distinguishing between bacterial and viral infections [9]. 

## 5. Distinguishing between Bacterial and Viral Infections with the Clinical Infection Score (CIS) Point [9, 10] 

To determine whether the diagnostic yield of measured individual variables increases upon combination, we estimated the clinical infection score (CIS) point consisting of four variables, including CRP (ROC curve cutoff point = 77 mg/L), ESR (28 mm/h), mean amount of CR1 on neutrophil (MFI of 8.7) and TNCR index (3.4). For every variable measured, a result less than the cutoff point was converted to a variable score point of 0, that between the cutoff point and an additional second cutoff value (161 mg/L for CRP, 42 mm/h for ESR, MFI of 13.5 for CR1 and 3.9 for TNCR index), was converted to a variable score point of 1, and that greater than the additional second cutoff point value was converted to a variable score point of 2 (Figure 2(a)). An additional second cutoff value of a variable was the maximum value detected in patients with viral infection. The maximum virus value of higher than the average value of bacterial infection (epidemic nephropathy, ESR of 112 mm/h) was ignored when additional second cutoff values were put in their places. We obtained CIS points that varied between 0 and 8 by combining variable scores (Figure 2(b)). At a cutoff point of >2, the CIS points differentiated between microbiologically confirmed bacterial infection (*n* = 46) and viral infection (*n* = 38) with 98% sensitivity and 97% specificity [9]. 

## 6. Distinguishing between dsDNA and ssRNA Virus Infections with the DNA Virus Score (DNAVS) Point [11]

**Figure 3:**
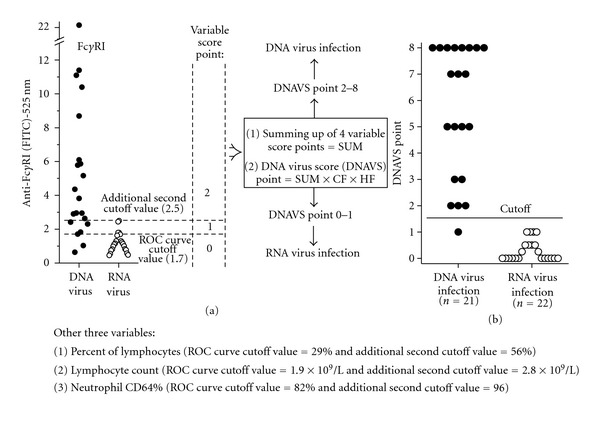
Formation of DNA virus score (DNAVS) point.

Similarly to CIS point, we estimated the DNA virus score (DNAVS) point consisting of four variables, including mean amount of CD64 on neutrophil (ROC curve cutoff point = MFI of 1.7), neutrophil CD64% (82%), percent of lymphocytes (29%), and lymphocyte count (1.9 × 10^9^/L). For every variable measured, a result less than the cutoff point was converted to a variable score point of 0, that between the cutoff point and an additional second cutoff value (MFI of 2.5 for CD64, 96% for neutrophil CD64%, 56% for percent of lymphocytes, and 2.8 × 10^9^/L for lymphocyte count) was converted to a variable score point of 1, and that greater than the additional second cutoff point value was converted to a variable score point of 2. An additional second cutoff value of a variable was the maximum value detected in patients with ssRNA virus infection (Figure 3). After data conversion, we obtained SUM that varied between 0 and 8 by adding four variable score points together. Next, we defined a DNAVS point by multiplying the SUM, CD64 factor (CF), and haematopoietic factor (HF) (DNAVS point = SUM × CF × HF). CF of 0.25 was used when variable score point of both receptor variables was 0. If variable score point of both haematopoietic variables was 0, then HF of 0.5 was used. In all the other cases, CF and HF were 1. At a cutoff point of higher than or equal to 1.5, the DNAVS points differentiated between dsDNA and ssRNA virus infections with 95% sensitivity and 100% specificity [11]. 

## 7. Distinguishing between Bacterial Infections, Viral Infections and Inflammatory Diseases with the Analysis of Fc**γ**RI Expression [12–14] 

The average number of Fc*γ*RI on the surfaces of both neutrophils and monocytes was significantly increased in patients with febrile viral and bacterial infections, compared to healthy controls. Furthermore, we describe a novel marker of febrile infection, designated “CD64 score point”, which incorporates the quantitative analysis of Fc*γ*RI expressed on both neutrophils and monocytes, with 94% sensitivity and 98% specificity in distinguishing between febrile infections and healthy controls. By contrast, analysis of Fc*γ*RI expression on neutrophils and monocytes displayed poor sensitivity (73% and 52%) and specificity (65% and 52%) in distinguishing between bacterial and viral infections, and the levels did not differ significantly between systemic (sepsis), local, and clinically diagnosed bacterial infections. Thus, the increased number of Fc*γ*RI on neutrophils and monocytes is a useful marker of febrile infection but cannot be applied for differential diagnosis between bacterial and viral infections or between systemic and local bacterial infections [12]. 

**Figure 4:**
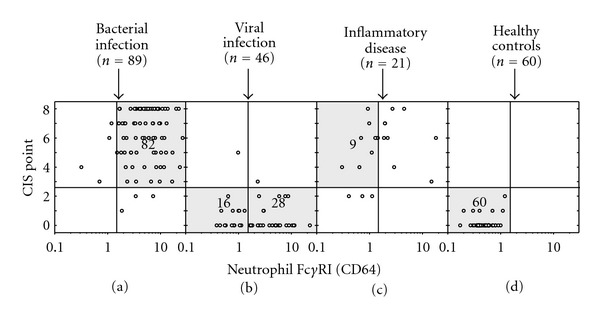
Fc*γ*RI/CIS point bivariate dot-plot graph.

As noticed above, the expression of neutrophil CD35 is higher in bacterial than in viral infections. Neutrophil CD35-based differentiation between bacterial and viral infections can be improved by generating the CIS point. We further developed CD64/CIS point bivariate dot-plot graph (Figures 4(a)–4(d)), where the vertical and horizontal lines are set to represent the optimal cutoff point, MFI value of 1.5 for neutrophil Fc*γ*RI and 2.5 for CIS point value, respectively. The bivariate dot-plot graph can be divided into four quadrants: upper left quadrant (ULQ), upper right quadrant (URQ), lower left quadrant (LLQ), and lower right quadrant (LRQ). Now, 92% of bacterial infections are located in URQ whereas viral infections are located in LLQ (35%) or in LRQ (61%). Inflammatory diseases distributed to LLQ (14%), ULQ (43%), and URQ (43%) [13].

## 8. Detecting Gram-Positive Sepsis [15]

In Gram-negative bacterial infection (*n* = 21), the average amount of CD11b on neutrophils was significantly higher than in gram-positive bacterial infection (*n* = 22). On the contrary, CRP level was significantly higher in Gram-positive than in gram-negative bacterial infection. By dividing the serum CRP value by the amount of CD11b on neutrophils, we derived a novel marker of Gram-positive sepsis, CRP/CD11b ratio, which displayed 76% sensitivity and 80% specificity for the detection of Gram-positive sepsis (*n* = 17) among febrile patients with microbiologically confirmed or clinically diagnosed bacterial infection. 

## 9. Conclusion

 Treating viral illnesses or noninfective causes of inflammation with antibiotics is ineffective and contributes to the development of antibiotic resistance, toxicity and allergic reactions, leading to increasing medical costs. A major factor behind unnecessary use of antibiotics is, of course, incorrect diagnosis. For this reason, timely and accurate information on whether the infection is bacterial in origin would be highly beneficial in the fight against antibiotic resistance. The analysis of the expression levels of Fc*γ*RI, CR1, and CR3, along with CRP and ESR data, provides a novel application to the diagnosis of infectious and inflammatory diseases. The best clinical benefit from the quantitative analysis of these markers will be obtained when the individual variables are combined to generate the CIS point method for a reliable bacterial infection marker, DNAVS point for differentiating between DNA and RNA virus infections, CD64 score point for a marker of febrile infection and CRP/CD11b ratio for a marker of gram-positive sepsis.

## Figures and Tables

**Table 1:** Monoclonal antibodies used in receptor expression studies.

Clone	Conjugate	Specificity	CD group	Isotype
Fc*γ*Rs				
22	FITC	Fc*γ*RI	CD64	IgG mouse
2E1	PE	Fc*γ*RII	CD32	IgG2a mouse
3G8	FITC	Fc*γ*RIII	CD16	IgG1 mouse

CRs				
J3D3	FITC	CR1	CD35	IgG1 mouse
Bear1	PE	CR3	CD11b	IgG1 mouse

Isotype controls				
679.1Mc7	FITC/PE	Irrelevant	—	IgG1 mouse
U7.27	PE	Irrelevant	—	IgG2a mouse

**Table 2:** Receptor expression changes in various diseases compared to healthy controls.

Receptor	Bacterial infection	Viral infection	Kidney cancer	Atopic dermatitis
Neutrophils				
CR1/CD35	+++	(−)	no change	+
CR3/CD11b	+++	+	++	(+)
Fc*γ*RI/CD64	+++	+++	(+)	no change
Fc*γ*RII/CD32	+	(−)	no change	(+)
Fc*γ*RIII/CD16	(−)	no change	no change	(−)

Monocytes				
CR1/CD35	+++	++	+	(+)
CR3/CD11b	+++	++	+++	(+)
Fc*γ*RI/CD64	+++	+++	+	(−)
Fc*γ*RII/CD32	(+)	no change	++	(+)

The +/− without parentheses indicates a significant increase/decrease in the expression of receptor in question compared to healthy control.

The +/− in parentheses represents an insignificant increase/decrease in the expression of receptor in question compared to healthy control.

+ or − = 0%–50% increase or decrease compared to healthy control, ++ = 50%–100% increase compared to healthy control, and +++ = more than 100% increase compared to healthy control.

**Table 3:** Parameters measured in the patient material expressed as mean (S.D.). Receptor expression data from both heparin and EDTA anticoagulated blood samples are presented.

Variables	Microbiologically confirmed		Healthy Control	Clinically diagnosed	
	Bacterial infection	Viral infection	(*n* = 60)	Bacterial infection	Viral infection
	(*n* = 46)	(*n* = 38)		(*n* = 43)	(*n* = 8)
CRP (mg/L)	232 (135)	40 (41)	—	217 (103)	43 (49)
ESR (mm/h)	65 (28)	19 (19)	—	69 (27)	22 (15)
WBC (×10^9^/L)	11 (4.9)	7.7 (4.1)	4.8 (1.3)	9.8 (4.8)	5.9 (1.4)
PMNL (%)	71 (14)	51 (22)	51 (9.8)	74 (13)	49 (20)
PMNL (×10^9^/L)	8.2 (3.6)	3.5 (2.0)	2.6 (0.9)	7.5 (3.9)	2.6 (1.0)

Heparin sample					
Neutrophil CR1	21 (9.9)	5.7 (2.9)	6.3 (2.2)	20 (7.5)	6.4 (3.3)
Neutrophil CR3	100 (51)	54 (23)	49 (18)	104 (45)	59 (35)
TNCR index	4.1 (0.5)	2.9 (0.5)	2.8 (0.3)	4.0 (0.5)	2.9 (0.5)
CIS point	6.2 (1.7)	0.6 (1.0)	—	6.3 (1.9)	0.6 (1.2)

EDTA sample	(*n* = 15)	(*n* = 6)	(*n* = 18)		
Neutrophil CR1	8.3 (2.4)	6.2 (2.8)	4.8 (1.3)	—	—
Neutrophil CR3	34 (12)	36 (11)	28 (6.0)	—	—
